# How are you doing in the eyes of your spouse? Level of agreement between the self-completed EQ-5D-5L and two proxy perspectives in an orthopaedic population: a randomized agreement study

**DOI:** 10.1186/s12955-021-01679-y

**Published:** 2021-01-27

**Authors:** Maria C. J. M. Tol, Jurrian P. Kuipers, Nienke W. Willigenburg, Hanna C. Willems, Rudolf W. Poolman

**Affiliations:** 1 Department of Orthopaedic Surgery, Joint Research, OLVG, P.O. Box 3075, 1000 WC Amsterdam, The Netherlands; 2 Department of Internal Medicine and Geriatrics, AMC, Amsterdam, The Netherlands; 3 Department of Orthopaedic Surgery, LUMC, Leiden, The Netherlands

## Abstract

**Objectives:**

To determine the level of agreement between both proxy versions and the self-completed EQ-5D-5L.

**Design:**

A randomized agreement study.

**Setting and participants:**

We recruited 120 patients (compos mentis) and their proxies at the orthopaedic outpatient clinic. Patients completed the regular EQ-5D-5L and their proxy completed the proxy version of the EQ-5D-5L and rated the patients’ health from their own (proxy-proxy) perspective (i.e. how do you rate the health of the patient), and from the patient’s (proxy-patient) perspective (i.e. how do you think the patient would rate their own health if they were able to).

**Measures:**

The primary outcome was the agreement between patients and their proxy, quantified as the intra class correlation coefficient for the EQ-5D-5L Utility score.

**Results:**

Average Utility scores were 0.65 with the self completed EQ-5D-5L, versus 0.60 with the proxy-patient version and 0.58 with the proxy-proxy version. The ICC was 0.66 (95% CI 0.523, 0.753) for the proxy-patient perspective and 0.58 (95% CI 0.411, 0.697) for the proxy-proxy perspective. The mean gold standard score of the VAS-Health was 69.7 whereas the proxy-proxy perspective was 66.5 and the proxy-patient perspective was 66.3.

**Conclusion and implications:**

The proxy-patient perspective yielded substantial agreement with the self completed EQ-5D-5L, while the agreement with the proxy-proxy perspective was moderate. In this study population of patients without cognitive impairment, proxies tended to underestimate the quality of life of their relative.

## Introduction

Due to the risen attention to value-driven care, studies to evaluate existing and new treatments are more common. The patient perspective is frequently at the center of focus during this healthcare evaluation. Health-Related Quality of Life (HRQoL) is an important patient reported outcome when comparing the effectiveness of health care interventions and the value of health care [1].

Scientists widely use the EQ-5D, which is known for its validity, reliability and responsiveness, as a measurement instrument for the HRQoL [2–5]. The EQ-5D is a descriptive system of health-related quality of life states consisting of five dimensions (mobility, self-care, usual activities, pain/discomfort and anxiety/depression) as well as a visual analogue scale (EQ VAS) on which patients rate their overall health.

The EQ-5D-5L is a questionnaire completed by the patient. Two by proxy versions were developed for patients who are not capable of completing the questionnaire by themselves due to i.e. cognitive impairment [6]. The difference between both proxy versions is the perspective of the answers. The instruction, how to fill in the questionnaire, is different: The first version is from the proxy’s perspective (how do you rate the patients health), the second version is from the patients’ perspective (how do you think the patient would rate their own health if they were able to do so).

Both versions are used more often, not only due to a growing group of elderly who are incapable of completing their own questionnaires, but also as a result of increased attention to value based health care and cost-efficiency research in which the patient perspective is often used [7–9]. Assessing the quality and effectiveness of treatments is challenging when patients, for instance with dementia, are incapable of understanding questionnaires. These developments strengthen the need for outcome assessment by proxy.

Never has it been investigated which by proxy version of the EQ-5D-5L is most likely to reflect the patient’s quality of life best [10–12]. We hypothesize that there might be a difference between the by proxy versions. Hence, it is important to know which proxy version best reflects quality of life in patients who can not complete the questionnaire themselves since it has a central role in healthcare evaluation research. Therefore, the aim of this study was to determine the agreement of both proxy versions with respect to the self reported quality of life using the EQ-5D-5L. This novel study will investigate the agreement of mentally healthy patients with their proxy.

## Methods

### Participants and setting

The study protocol was approved by the medical ethics committee at OLVG (WO 18.059) and conducted according to the principles of the Declaration of Helsinki, as amended in Seoul and Fortaleza (64th WMA General Assembly, October 2013). The trial was registered at The Netherlands National Trial Register (TC 7526). To ensure that self-completed questionnaires could be used as gold standard, the study population consisted of mentally healthy patients. And to accurately reflect a typical clinical setting, their relatives or friends that accompanied them to the outpatient visit were asked as proxy. We asked all consecutive patients of both sexes and all ethnicities who visited the orthopaedic outpatient clinic accompanied with a proxy during the inclusion period, June 2018–August 2018, to participate in our study. Patients were eligible for study participation when the following inclusion criteria were met: 18 years or older at the time of visiting the outpatient clinic (both patient and proxy giver), compos mentis, Dutch fluency and literacy. We excluded patients when they had signs of cognitive impairment or when they visited the outpatient clinic alone. Any signs of cognitive impairment were objectified by a clinician.

### Study procedures

The EQ-5D-5L descriptive system assesses health in five dimensions (mobility, personal care, usual activities, pain/discomfort, and anxiety/depression), each of which has five levels of response (no problems, slight problems, moderate problems, severe problems, extreme problems/unable to). This part of the EQ-5D questionnaire provides a descriptive profile that can be used to generate a health state profile. Health state index scores generally range from less than 0 (where 0 is the value of a health state equivalent to dead; negative values representing values as worse than dead) to 1 (the value of full health), with higher scores indicating higher health utility. The second part of the questionnaire consists of a visual analogue scale (VAS) on which the patient rates his/her perceived health from 0 (the worst imaginable health) to 100 (the best imaginable health.

There are two proxy perspectives of the EQ-5D-5L. The questionnaire and scoring are the same as the self completed EQ-5D-5L. The instruction, how to fill in the questionnaire, is different: The first version is the proxy-proxy perspective: (how do you rate the patients health) the proxy is asked to rate the patients’ health related quality of life in their (the proxy’s) opinion. The second version is the proxy-patients perspective (how do you think the patient would rate their own health if they were able to do so) the proxy is asked to rate how he/she (the proxy) thinks the patient would rate his/her own health-related quality of life if the patient were able to communicate it.

**Fig. 1:**
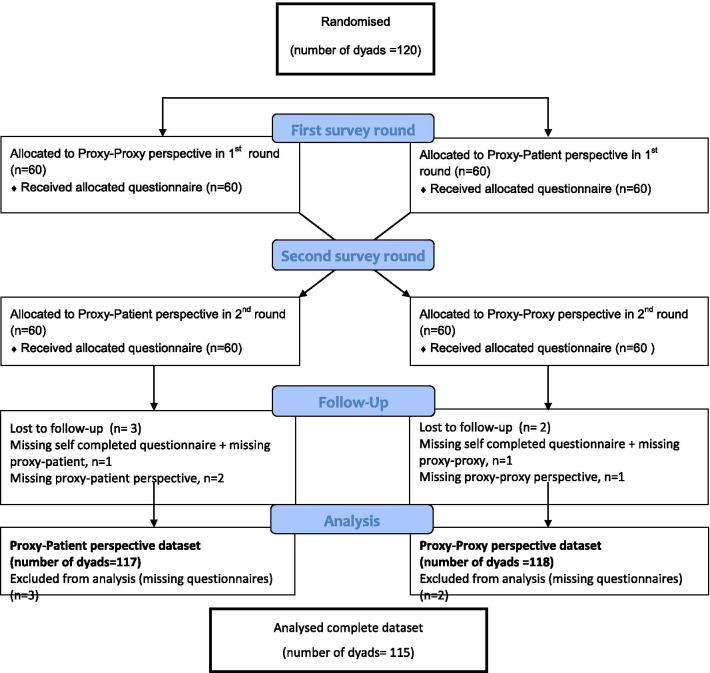
Study design and flowchart

All patients received the self-complete version of the EQ-5D-5L, resulting in the gold standard, and all proxy givers completed both proxy versions. Dyads were randomly assigned to which order they the proxy had to complete the two different questionnaires in a 1:1 allocation ratio, using variable block randomisation in CASTOR EDC (specs) (Fig. 1). Prior to participation, the patients and their proxies were not aware of the existence of the different perspectives of the proxy version and were blinded for the purpose and hypothesis of our study. Proxies were only given the second version upon completing the first. Patient and proxy were asked to complete the questionnaire separated from their proxy to assure they answered independently.

### Statistical analysis

We converted the scores on the five dimensions of the EQ-5D-5L into the Utility score, which is reflecting the HRQoL. We used the Euroqol EQ-5D-5L Crosswalk Index Value Calculator for calculating Utility scores derived from the Dutch general population [13]. A higher score indicates a higher rated quality of life. To quantify the level of agreement between the (continuous) Utility score of the Self-complete EQ-5D-5L and their proxy versions, we used the Intraclass Correlation Coefficient (ICC), based on absolute agreement in a two-way mixed effects model. We repeated this analysis for the EQ-VAS score. In addition, we performed Weighted Kappa and absolute agreement analysis to quantify the level of agreement for the (categorical) individual domain scores. For the interpretation of both ICC and Weighted Kappa we used the method of Landis and Koch, with scores > 0.81 indicating almost perfect agreement, 0.61–0.8 substantial, 0.41–0.60 moderate, 0.21–0.4 fair and < 0.20 slight agreement [14]. The level of significance was < 0.05. To assess whether the health status influences the accuracy in which the proxy giver can assess the patient’s health status, we visualized the difference between the self-completed Utility scores and both proxy perspective Utility scores with respect to the self completed EQ-5D-5L Utility scores. We performed all analyses using IBM SPSS Statistics (Version 22.0). Since there was no effect size available to analyse an adequate sample size, the sample size was based on previous studies on this topic [11, 15].

## Results

### Population

We enrolled a total of 120 dyads, consisting of 120 patients and their proxies. Between May and July 2018 we received 115 complete and 5 incomplete datasets of the self-completed and their proxy perspectives for data analysis. There was one missing self-completed questionnaire, 1 missing proxy-proxy perspective, 2 missing proxy-patient perspective and 1 missing questionnaire of both perspectives. The baseline characteristics of the patients and proxy givers are depicted in Table 1.

**Table 1:** Baseline characteristics of patients and proxy givers

Patient gender (total n = 120), n (%)	
Female	74 (61.7)
Proxy gender, (total n = 120) n (%)	
Female	62 (51.7)
Patient age group, n (%)	
18–45 years	29 (24.2)
45–70 years	51 (42.5)
≥ 70 years	40 (33.3)
Proxy age group, n (%)	
18–45 years	23 (19.2)
45–70 years	75 (62.5)
≥ 70 years	22 (18.3)
Relationship to patient, n (%)	
Partner	71 (59)
Sibling	5 (4.2)
Child	15 (12.5)
Parent	15 (12.5)
Friend	9 (7.5)
Neighbour	1 (0.8)
Other	4 (3.3)

*n* Number of patients or proxies

### Absolute scores

The mean Utility score of the patients self completed EQ-5D-5L (gold standard) was 0.65 (95% CI 0.614, 0.686), of the proxy-proxy perspective 0.58 (95% CI 0.538, 0.621) and of the proxy-patient perspective 0.60 (95% CI 0.562, 0.638). The mean gold standard score of the VAS-Health was 69.7 (95% CI 66.4, 73.1), whereas the proxy-proxy perspective was 66.5 (95% CI 63.1, 69.8) and the proxy-patient perspective was 66.3 (95% CI 62.8, 69.8) (Table 2).

**Table 2:** Mean scores EQ-5D and VAS Health

	Patients (gold standard) number of patients = 119	Proxy–proxy perspective number of dyads = 118	Proxy–patient perspective number of dyads = 117
Utility score EQ-5D-5L mean (95%CI)	0.65 (0.614–0.686)	0.58 (0.538–0.621)	0.60 (0.562–0.638)
	n = 117	n = 118	n = 115
VAS health mean (95%CI)	69.74 (66.4–73.1)	66.48 (63.1–69.8)	66.33 (62.8–69.8)

*SD* standard deviation

### Agreement scores

For the Utility score, the proxy-patient perspective had a substantial level of agreement with the gold standard: ICC 0.66 (95% CI 0.523, 0.753). The proxy-proxy perspective had a moderate level of agreement with the gold standard: ICC 0.58 (95% CI 0.411, 0.697) (Table 3). For the overall health status based on the EQ-VAS, the level of agreement was substantial with the proxy-patient perspective: ICC = 0.64 (95% CI 0.515, 0.737), versus a moderate level of agreement of proxy-proxy perspective: ICC = 0.53 (95% CI 0.386, 0.65) (Table 3).

**Table 3:** Level of agreement utility score and VAS-Health

	Proxy–proxy perspective number of dyads = 115	Proxy–patient perspective number of dyads = 115
Utility score		
ICC (95% CI)	0.58 (0.411–0.697)	0.66 (0.523–0.753)
Strength of agreement	Moderate	Substantial
VAS-health		
ICC (95% CI)	0.530 (0.386–0.650)	0.639 (0.515–0.737)
Strength of agreement	Moderate	Substantial

The level of agreement, measured with the Weighted kappa, of the individual domain scores are listed in Table 4. No differences in level of agreement in all subdomains were observed between the two proxy versions compared with the gold standard.

**Table 4:** Level of agreement domain scores EQ-5D-5L

	Proxy-proxy perspective number of dyads = 115		Proxy-patient perspective number of dyads = 115	
	Weighted kappa (SE)	95% CI	Weighted kappa (SE)	95% CI
Mobility	0.596 (0.053) moderate	0.492–0.699	0.599 (0.054) moderate	0.494–0.705
Selfcare	0.452 (0.065) moderate	0.324–0.579	0.417 (0.073) moderate	0.275–0.559
ADL	0.337 (0.061) fair	0.216–0.457	0.384 (0.061) fair	0.264–0.503
Pain	0.392 (0.070) fair	0.255–0.529	0.394 (0.068) fair	0.261–0.527
Anxiety	0.355 (0.066) fair	0.225–0.484	0.406 (0.073) fair	0.262–0.549

*n* Number of patients, *SE* standard error, *CI* confidence interval

Absolute agreement for individual domain scores (Table 5) was equal between proxy versions for the domain Selfcare. Absolute agreement was higher for the proxy-patient perspective in Mobility (2.5%) and Anxiety/depression (7.5%). Absolute agreement was higher for the proxy-proxy perspective in ADL (1.7%) and Pain (1.7%).

**Table 5:** Absolute agreement domain scores EQ-5D-5L

Domain	Proxy–proxy perspective (A) n = 115 (%)	Proxy–patient perspective (B) n = 115 (%)	Difference in agreement between perspectives	95% CI of difference
Mobility	55.8	58.3	A 2.5% < B	− 9.887, 14.780
Selfcare	60	60	0%	− 12.218, 12.218
ADL	39.2	37.5	A 1.7% > B	− 10.466, 13.796
Pain	52.5	50.8	A 1.7% > B	− 10.783, 14.108
Anxiety	51.7	59.2	A 7.5% < B	− 5.012, 19.688

**Fig. 2:**
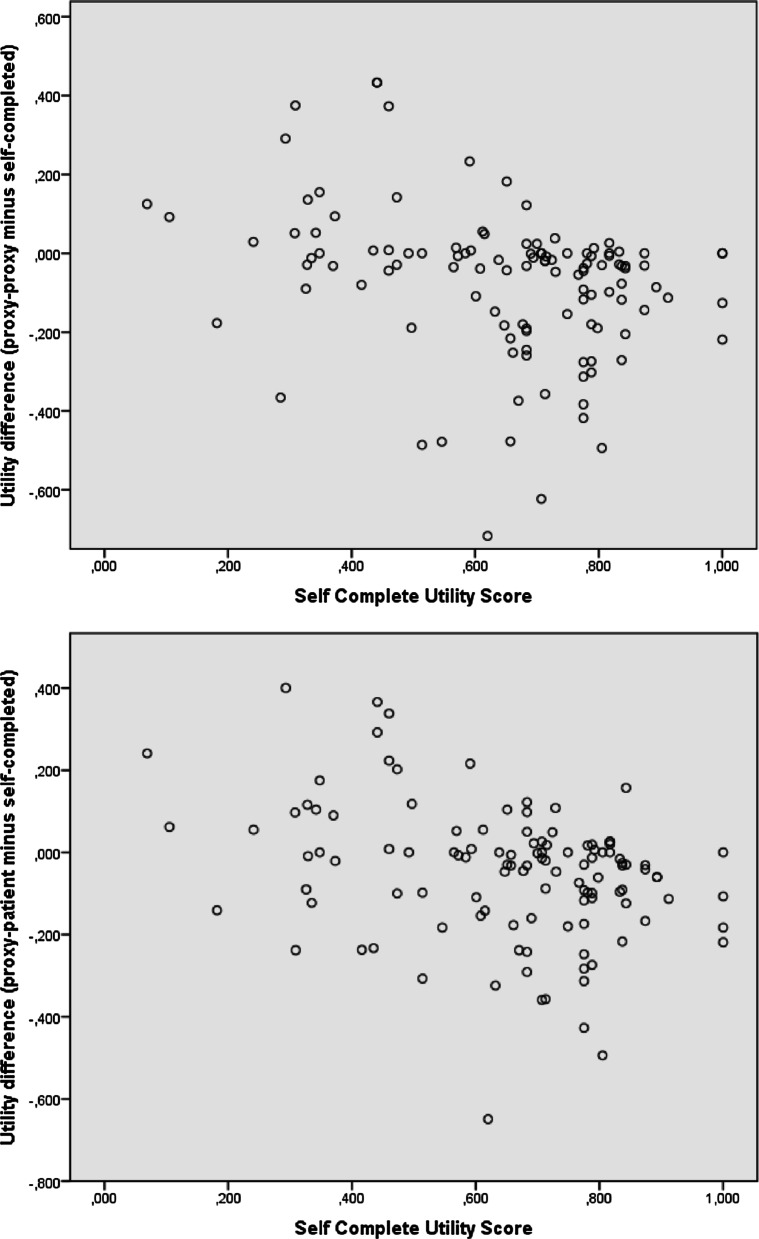
Evaluation of effect patients’ health status on proxies ability to accurately assess patient’s health status

Figure 2 shows that the discrepancy between Utility scores between proxy givers and patients does not depend on the patient’s health status (expressed in Utility score).

## Discussion

Our study showed substantial agreement between the patient gold standard and the proxy-patient perspective and moderate agreement between the patient gold standard and proxy-proxy perspective for both the Utility score and VAS Health of the EQ-5D-5L. The Utility score is the primary method to interpret health status using the EQ-5D-5L questionnaire. The mean Utility score was higher in the patient gold standard compared with both proxies.

This is the first study which empirically compares the two proxy perspectives of the EQ-5D-5L. Another strength of the study is that both proxy perspectives, given by one proxy, were compared with the same patient self-completed EQ-5D-5L questionnaire, resulting in adequate comparison. Moreover, the study design ruled out recall bias and participants were blinded to the study hypothesis.

All participants were Orthopaedic patients, which could be a sub-selection of the population and may be a limitation of the study. However, patient acquisition occurred in the waiting room at the outpatient clinic of all sub specializations of the Orthopaedic surgery where there is a high variance in the patient population. Due to this wide scatter we think the included patients and proxies can be a good representation of the population. The results of our study should however be evaluated in other disciplines. To ensure the self-complete EQ-5D-5L could be used as gold standard, our study population only included mentally healthy patients. Caution should be used when extrapolating the results of this study to a population with cognitive impairments.

We found one previous study that compared the two different proxy perspectives completed by a clinician with the self-completed EQ-5D with three levels (3L) [15]. That study reported substantially higher levels of agreement for all domains and utility score compared with our results. They reported Kappa values > 0.7 on all individual domain scores, while our highest kappa was 0.6 (Mobility domain). This is likely explained by the fact that the EQ-5D-3L has only three answer categories per domain, opposed to five categories in the EQ-5D-5L. Therefore the potential for disagreement is higher for the EQ-5D-5L, resulting in lower levels of agreement for domain scores and eventually Utility score.

All proxy questionnaires where completed by relatives of, or persons close to, the patients in this study. The observed agreement for individual domain scores supports previous findings on higher validity of questionnaires provided by relatives than clinicians for the less observable dimensions (‘Anxiety/depression’ and ‘Pain’) [11, 16]. However, Bryan and co-investigators showed that data provided by clinicians had higher construct validity regarding more observable dimensions of the EQ-5D-3L instrument (e.g. ‘Mobility and Selfcare’) [11]. Understandably, clinicians are more experienced than relatives at objectively assessing patient functioning as part of routine clinical assessment and have a wide range of knowledge regarding loss of function. Relatives on the other hand are far better acquainted with the patient and therefore more able to empathize with their subjective reality. However, it is remarkable that proxies underestimate the self-related health status of the patient. This phenomenon is observed in more studies, but non of the authors has given an explanation for the observed effect [10–12]. One explanation could be that the effect is influenced by the proxies own mood, personal beliefs or expectations [17]. Another explanation why elderly overestimate their health status could be that they compare their own health status with peers suffering from worse health, which give them a positive perception of their own function [18].

The effect of the underestimation of the health status by proxies is important for clinicians. This means that treatment decisions based on proxy perspectives could be based on a health status worse than the real health status. On the other hand if patients overestimate their health status, it could be associated with riskier health behavior [19].

Further research could be focused on optimizing the level of agreement between the proxy perspective and the gold standard. For example by completing the observable dimensions of proxy questionnaires through clinicians and the subjective dimensions through relatives. In addition, despite the barriers of cognitive limitations, further research should be done in patients with mild to moderate stage dementia to validate the proxy version for this specific patient group.

## Conclusion

This study showed a substantial agreement between the patient gold standard and the proxy-patient perspective and moderate agreement between the patient gold standard and proxy-proxy perspective for both the Utility score and VAS Health of the EQ-5D-5L. Therefore we recommend the proxy-patient version of the EQ-5D-5L. Regardless of their perspective, in this study population of patients without cognitive impairment, proxies tended to underestimate the quality of life of their relative.
